# Algorithms for Reconstruction of Impedance Spectra from Non-uniformly Sampled Step Responses

**DOI:** 10.2478/joeb-2022-0020

**Published:** 2023-01-14

**Authors:** Y. Zaikou, C. Gansauge, D. Echtermeyer, U. Pliquett

**Affiliations:** 1 Institute for Bioprocessing and Analytical Measurement Techniques, Heilbad Heiligenstadt, Heilbad, Germany

## Abstract

Fast and reliable bioimpedimetric measurements are of growing importance in many practical applications. In this work we used a measurement method in time domain by processing the step response of the biological system under test. In order to decrease the data volume and computation time while retaining all relevant information the step response is sampled non-uniformly. Consequently, fast Fourier transform cannot be directly used for spectrum calculation and non-conventional data processing algorithms for transforming measured data into the frequency domain are required.

In this paper we present corresponding computational methods. They are split into two groups. The first group is oriented on calculating the local approximation of the measured step response with a set of proper functions and calculating its spectrum via analytical Fourier transform, thus yielding a relatively versatile approach for estimating the impedance spectrum. In this case, the choice of approximating functions that suit known a priori properties of the measured signals are of great importance.

A second group of methods relies on the evaluation of important signal parameters directly in the time domain. In this case we use a priori information about the measurement object in the form of an underlying model. After that the model is fitted to the measured data and thus, parameter values are extracted.

Practical aspects, advantages and drawbacks of all considered data processing steps are revealed when applying them to the measurements made with real biological objects.

## Introduction

The passive electrical characterization of materials, including biological materials such as cells, cell suspensions or tissue, is generally understood in terms of impedance measurements. Classically, the instrument sweeps through a frequency range and measures the impedance at each selected frequency. This procedure takes time and thus it is not applicable for monitoring fast changes of the object state. Moreover, it requires expensive equipment with high power consumption.

A faster method is based on monitoring the system response to broadband excitation. Although a direct measurement in the time domain, assessing the relaxation due to a transient stimulus like a step or Dirac function, is known and used since decades, it is rarely used in bioimpedance measurements. The main reason is the wide availability of impedance analyzers, but also the rejection of a less known method.

In recent years, the time-domain method started to gain popularity, especially in process control instrumentation. However, while the measurement is done in the time domain using a broad bandwidth stimulus, further processing takes place in the frequency domain after Fourier transformation of the measured signals. In this case, a huge data volume needs to be recorded and processed, especially in broadband measurements. According to signal theory, each decade of bandwidth multiplies the data volume by two orders of magnitude, resulting in, for instance, 2 million data points for a signal with a bandwidth between 100 Hz and 100 MHz. Taking one measurement even with a size of several million points is feasible but becomes problematic if continuous monitoring over several orders of magnitude in frequency is required. A way out is the use of step functions as stimulus and tracing the response, which is in case of biological objects fast changing at the beginning but levels off with longer time. In this case the data volume can be reduced by performing non-uniform sampling, not to be confused with data compression being the classical application of nonuniform sampling [8]. However, non-uniform sampling requires non-conventional spectrum calculation since simple Fourier transformation is impossible for non-equidistantly spaced vectors. Fourier transformation means the integration of the signal multiplied with the known function. Thus, a straightforward solution would be in using the numerical integration over every two subsequent sampling intervals, for example, according to the well-known Simpson rule. In practice, however, this approach yields artifacts in the frequency domain since it disrespects important properties of the underlying signal model like its smoothness. These artifacts become very significant when the signal is measured at only a few instants. For example, this is the case in extremely low power solutions with minimalistic hardware. Such a system was previously implemented and described in [5].

In this work we show different approaches for transforming the non-equidistantly sampled step response into the frequency domain while avoiding artifacts. The basic idea is to employ preexisting knowledge about the object and rejections of any solution unlikely for this particular object.

## Materials and methods

**Fig. 1:**
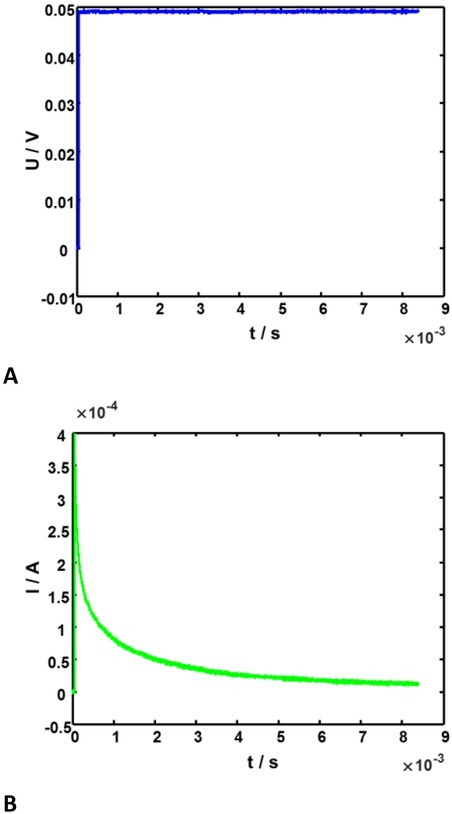
Example of stimulus and response signals. **A**: Stimulus **B**: Step response.

Without the loss of generality, we can point out the following properties of the step response of cell suspensions or tissues, both properties being observable in figure 1:

- The current that represents the system response to a voltage step decreases monotonously, fast immediately after the step application but slowing down with time.
- The early time of the step response corresponds to high frequency part of the spectrum.

These a priori facts about our signals extensively influence the design of measurement system and corresponding data processing.

From the viewpoint of signal processing, it makes perfect sense to sample faster when we expect higher frequencies to be present. On the other hand, sampling the complete response with high sampling rate is demanding and generates huge data volumes that cannot be processed within reasonable time employing limited resources. This leads to the idea of using non-equidistant sampling, faster at the early-time part of the measured signal and slowing down towards later time and, accordingly, towards lower frequencies [1].

Since the step response of biological objects is often well represented as a sum of exponentials [2], logarithmically spaced sampling can be considered as natural. In order to avoid aliasing and decrease noise contribution, especially in late time, logarithmic sampling is performed via integration. That is, we do not really measure the current, rather its integral as voltage at an integrating capacitor where sampling instants $t_{i}$are spaced logarithmically:


(1) $$U_{C}=\frac{1}{C}\int_{t_{0}}^{t_{i}}I(t)dt$$

This means that in order to calculate impedance via Ohm’s law one needs to obtain the derivative of the acquired signal first. A simplistic numerical estimate and the corresponding sampling time vector is given by the following equations:


(2) $$\bar{I_{i}}=I\left(t_{s}\right)=C\frac{U\left(t_{i}\right)-U\left(t_{i-1}\right)}{t_{i}-t_{i-1}};t_{s}=\frac{t_{i}+t_{i-1}}{2}$$

**Fig. 2:**
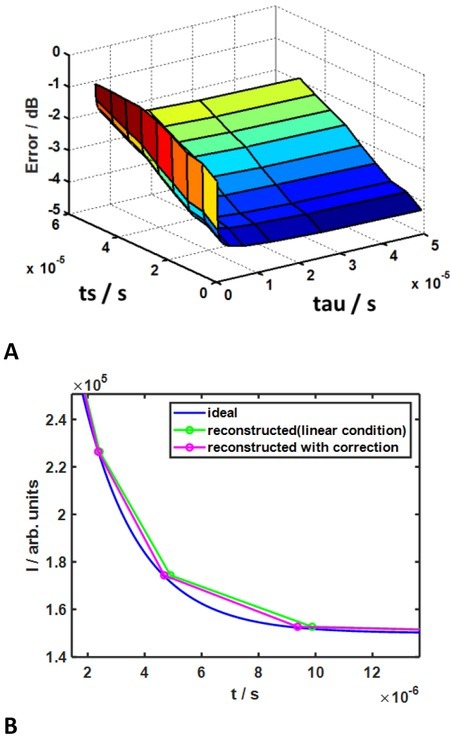
Error of simplistic sampling instant estimation and its correction. **A**: Dependency on time constant of exponential signal and on sampling interval. **B**: Example of correcting the error.

Here we suppose that the numerical derivative of the measured signal appears exactly at the time instants in the middle between the measured samples, which implies a linear charging of the integration capacitor with time or a steady current at the time basket under consideration. However, such an estimate can be imprecise in case fast signals are sampled slowly which yields a relative error distribution as shown in figure 2, A.

With increasing departure from steady current, $\frac{dU_{C}}{dt}\neq$ *const* equation 2 where we implicitly suppose linear charging of the integration capacitor between sampling instants, becomes invalid. Thus, a better approximation of the measured signal is given, for example, by polynomial of third order. Polynomial coefficients can be calculated from the system of linear equations:


(3) $$\begin{array}{c} A_{i}t_{i-1}^{3}+B_{i}t_{i-1}^{2}+Ct_{i-1}+D=u_{i-1}\\ A_{i}t_{i}^{3}+B_{i}t_{i}^{2}+Ct_{i}+D=u_{i}\\ A_{i}t_{i+1}^{3}+B_{i}t_{i+1}^{2}+Ct_{i}+D=u_{i+1}\\ A_{i}t_{i+2}^{3}+B_{i}t_{i+2}^{2}+Ct_{i+2}+D=u_{i+2} \end{array}$$

According to the definition of the average value of a function, we find for the current


(4) $$\bar{I_{i}}=\frac{1}{t_{i}-t_{i-1}}\int_{t_{i-1}}^{t_{i}}\left(3A_{i}t^{2}+2B_{i}t+C\right)dt$$

Since the average value of a function is certainly reached at an unknown instant on corresponding time interval, it follows from equation 4 that sampling instants of the first derivative can be estimated as solutions of the following problem:


(5) $$\bar{I_{i}}=3A_{i}t^{2}+2B_{i}t+C$$

Equation 5 yields multiple solutions. However, since $\bar{I_{i}}$ and its approximation is monotonous, which is expected for our measured signal, only one solution belongs to the interval $\left[t_{i-1},t_{i}\right].$This solution represents the actual sampling instant $t_{s}$ and its knowledge allows us to calculate impedance spectrum more precisely.

An important step to be taken before calculating the impedance spectrum according to Ohm’s law, is Fourier transformation of the local approximation of the differentiated signal:


(6) $$F(I(t))=A\left(\omega_{k}\right)=\int_{t_{0}}^{t_{n}}I(t)e^{-j\omega_{k}t}dt$$

In absence of an analytical solution, discrete calculation yields:


(7) $$A\left(\omega_{k}\right)=\sum_{i=2}^{N}\int_{t_{i-1}}^{t_{i}}f_{i}(t)e^{-j\omega_{k}t}dt$$

Fourier transformation itself can be performed analytically or, when corresponding integrals cannot be solved, by means of discrete Fourier transform, preferably with a fast Fourier transformation algorithm.

**Fig. 3:**
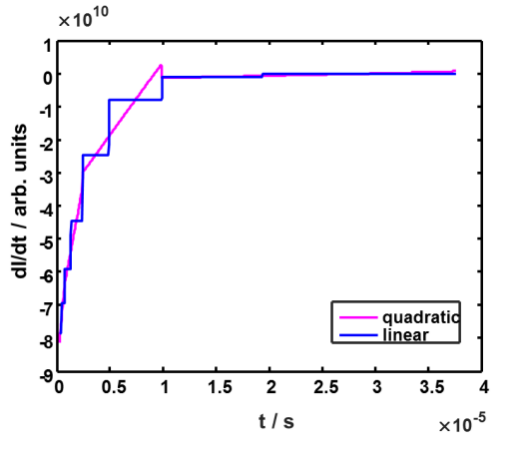
Derivatives of the straightforward local approximations.

As for the local approximations of current $f_{i}(t),$straightforward approaches when $f_{i}(t)$are calculated independently from one another, suffer from serious drawbacks. Essentially, discontinuities of the derivative of the measured signal or, sometimes, of the signal itself, are likely to arise at the sampling instants (figure 3).

Even though it appeared to be possible to alleviate the influence of such discontinuities by means of filtering, we compare different methods yielding continuity at the sampling points.

Most common of them include: linear approximations, cubic splines [7] and other polynomial splines that do not take into account any a priori information about our signals. All these methods possess some advantages. Linear approximation $f_{i}(t)=ct+d$is fast (which is important in our task) and simple.

In order to calculate the spectrum according to eq.7 we need to know the Fourier coefficients. In case of linear approximation, they are given by:


(8) $$\int f_{i}(t)e^{-j\omega_{k}t}=\frac{e^{-jt\omega_{k}\omega_{k}\left(jc\omega_{k}t+jd\omega_{k}+c\right)}}{\omega_{k}^{2}}+\text{ const }$$

**Fig. 4:**
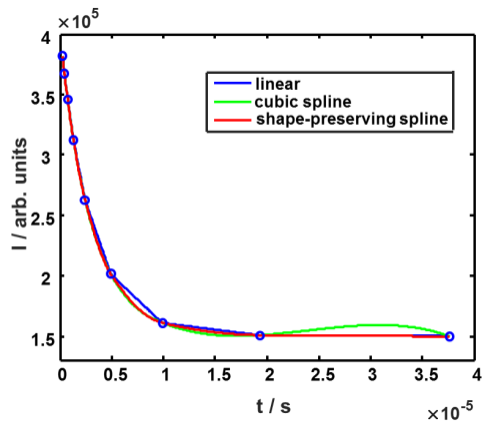
Local signal approximations used to reconstruct measured signal.

Cubic spline is the smoothest third order polynomial approximation possible, which has the advantage of a smooth underlying model that is well suited for objects like cells or biological tissue since for these objects a smooth underlying model is supposed. Nevertheless, disadvantages are serious. Our signals are essentially nonlinear as a function of time, but they do not change according to polynomial function between sample points. Therefore, cubic splines often produce unsatisfactory results like overshoot and oscillations with respect to reconstruction phenomena. These phenomena, observable in figure 4 are considered as artifacts that reduce the significance of the results since they deviate from the underlying theoretical models.

Shape-preserving splines introduced in [3] perform somewhat better. Their main advantage is that they guarantee monotonicity of the output signal if the input is a sampled signal monotonous at any point in time. Such behavior corresponds to a priori information about our signals. By using shape-preserving splines instead of simple cubic splines we avoid oscillations (figure 4, green curve). Fourier coefficients for shape-preserving spline and any other third-order approximation $f_{i}(t)=at^{3}+bt^{2}+ct+d$are given by:


(9) $$\begin{array}{r} \int f_{i}(t)e^{-j\omega_{k}t}=\\ \frac{e^{-jt\omega_{k}\left(j\omega_{k}^{3}(t(t(at+b)+c)+d)+\omega_{k}^{2}(t(3at+2b)+c)-2j\omega_{k}(3at+b)-a\right)}}{\omega_{k}^{4}}+\\ const \end{array}$$

One of the techniques that in many cases helped to improve the performance of the abovementioned approximations is the transformation of the time axis. From general considerations, it is likely that rescaling the timeline to emphasize the essential part can improve the approximation. In the ideal case, the measured signal becomes a linear function, and its approximation is trivial. In the same manner we can also expect that higher order approximations become more precise, since the error estimate is usually determined by the higher order derivatives and those are expected to decrease.

It should be noted that non-uniform sampling with a logarithmically spaced time vector applied in our work is already a rescaling of the timeline, which improves the sensitivity distribution of the signal. The amplitude of a stimulus square wave falls off with 1/n, where n is the number of the harmonics, which means that the sensitivity towards high frequency diminishes fast. This decrease in sensitivity is alleviated by denser sampling at the fast-changing part of the step response. Thus, sensitivity becomes smoothly distributed over the frequency range of interest.

In addition to such non-uniform sampling, we considered our measured signal in “logarithmical” time (ln⁡(t))and in ‘’exponential’’ time $\left(\exp\left(-\frac{t}{\tau_{r}}\right)\right).$In the particular case of the measured signal being single exponential with an offset and time constant $\tau_{r}$using local linear approximation in ‘’exponential’’ time would suffice: the signal is ideally described by a linear function and can be reconstructed without artifacts.

**Fig. 5:**
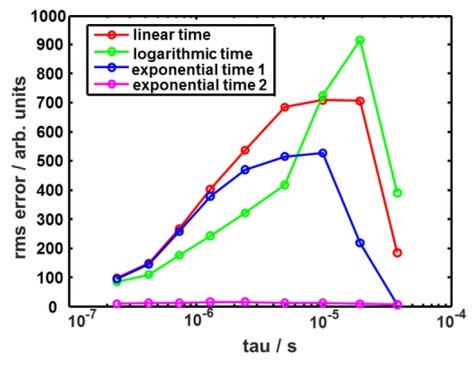
Simulated errors of time rescaling for single exponential signals. Reconstruction of measured signals is done by shape-preserving splines.

Simulated errors of time rescaling for single exponential signals with different time constants within our measurement range, are shown in figure 5.

It appears that ‘’logarithmic’’ time is the most efficient approximation for the fastest exponentials. However, for slower signal it performs even worse than linear time without any rescaling. As for the ‘’exponential time’’, we considered two particular cases. In the first case (‘’exponential time 1’’) the time constant of ‘’rescaling’’ was set to the measurement interval. In this case performance improvement due to rescaling for all time constants of the measured signals was reached in comparison with the case of no rescaling. In another case (‘’exponential time 2’’) we ‘’rescaled’’ with the time constant equal to the time constant of the measured signals. According to what was told above in this case the error approached zero. One can expect that if in a particular measurement scenario a better a priori guess can be made about the relaxation process of interest, than we made in case of ‘’exponential time 1’’, the reconstruction error will decrease until it reaches values of ‘’exponential time 2’’ in figure 5 if our guess was ideal.

When measured data is collected fast (like thousand spectrums per second) the monotonicity condition is not always fulfilled due to random noise. Certainly, this effect can be alleviated by averaging. However, simple averaging has drawbacks:

- We want to detect fast changes of the measurement object. When averaging, we lose information about these fast changes. Moreover, when averaging the signals measured at a continuously changing object, we can disturb our signal in a very unpredictable way. Finally, it becomes impossible to assign the actual measurement with the state of an object.
- Averaging itself does not take into account a priori information about the measured object (the above-mentioned monotonicity of current and its first derivative).

Thus, one can suppose that certain data processing techniques that take into account a priori information can outperform averaging. A method that seemed to produce the best results included the following steps:

- Finding the longest sequence composed of measured current samples that satisfies the a priori conditions (trusted samples).
- Discarding all samples that do not satisfy the a priori conditions and replacing them with values that are interpolated from the trusted samples.

Another way to calculate the impedance spectrum is to determine the parameters of theoretical step response of the object under test first. After this, the impedance spectrum can be calculated in the same way as with local approximations.

In general, the parameters of interest can be calculated via a nonlinear optimization procedure. Many algorithms of nonlinear optimization are extensively used in classic impedance spectroscopy [6]. In our case, however, the model is fitted in the time domain when the optimization problem is formulated as below.


(10) $$\arg\min_{x}F(x,t);\;F(x,t)=∥I(x,t)-f(x,t){∥}^{2}$$

Here x is the vector of the optimization parameters that we want to find out, f(x,t)is the model of measured step response I(x,t)⋅F(x,t)is called the objective function that we want to minimize and in the simplest case it is just the norm of the difference between measured signal and its theoretical model.

Straightforward nonlinear fit is cumbersome for online implementation. Other challenges of this approach are quite different from those of local approximations. First of all, relatively weak non-idealities of the measured signal (due to noise, etc.) can cause the convergence of a model to a completely wrong solution. On the other hand, continuity of the model, its monotonicity and the monotonicity of its derivatives are guaranteed by design. With respect to fitting the model, the significances of the derived parameters and calculation time are improved by the following steps:

- Putting more weight on the objective function of the time samples with less noise. There is a certain freedom in determining the objective function. Actually, it can be any function that reaches its global minimum when our model coincides with the measured signal. In case of noise-free data the objective function for optimization can look like it is shown in eq. 10. Given that we sample logarithmically it means putting more weight on the earlier time where we expect more useful information about our object to appear. However, we can also take into account that for the various reasons connected with the measurement object and electronics random noise is not homogeneous at different samples. Thus, in case of noisy data when calculating objective function one can normalize it with noise standard deviations:

(11) $$F(x,t)=∥\frac{I(x,t)-f(x,t)}{\sigma (t)}∥$$

By doing so we put more weight on the data samples which are less noisy and thus minimize the negative influence of random perturbations. From the viewpoint of detection theory such summation represents the optimal data processing method for the task when a signal needs to be detected within several samples and the signal to noise ratio (SNR) is different within these samples. Noise standard deviation within different samples can be easily evaluated via a short calibration measurement.

- Finding the parameter values that appear most often within some confidence interval. In the noisy measured data, many fits can yield completely wrong values. However, parameters will tend to group around the “correct” value. We can check the values and intervals where the fit parameters are grouping.
- Setting reasonable constraints for the fit parameters in case constrained minimization is preferred. Intervals where fit parameters can physically appear are known a priori and one can make use of this knowledge.
- Determining as many model parameters without fit as possible. When applicable, this step greatly increases both the calculation speed and measurement significance.
- It is advantageous to substitute one global fit with two or more local ones when a certain process is expected to be better observable at a certain time (e.g early time, late time). By doing so we determine the object parameters in the area where they manifest themselves strongest and reduce computation time.

## Informed consent

Informed consent has been obtained from all individuals included in this study.

## Ethical approval

The conducted research is not related to either human or animal use.

## Results and discussion

Let us consider the described approaches with respect to how they perform when applied to the measurement example. The measurement example represents the impedance spectrum of a spheroid passing through the glass nozzle in an electrolyte medium (impedance measurements with spheroids are considered in details in [4]).

**Fig. 6:**
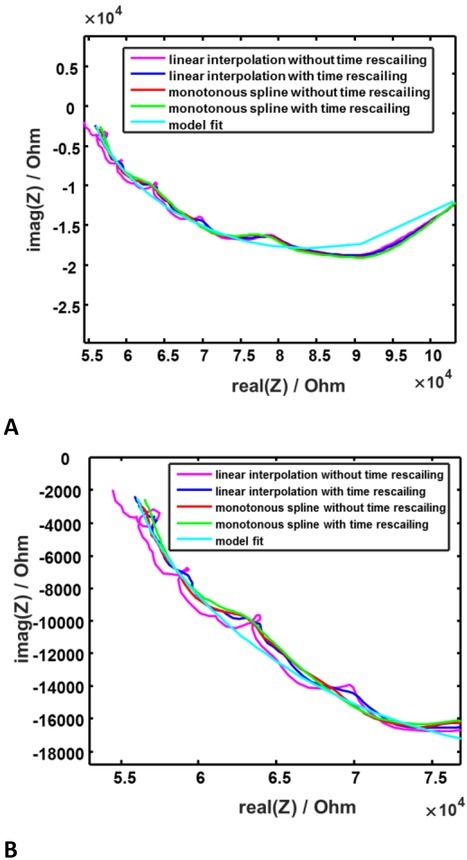
Local signal approximations with and without time rescaling for spheroid in nozzle. **A**: Whole spectrum. **B**: Zoom at higher frequencies.


The corresponding impedance spectrum is shown in figure 6. It can be seen that shape-preserving spline makes the local approximation smoother, especially in the challenging area of higher frequencies. The same is true for the time rescaling. The positive effect of time rescaling is more significant for linear approximation, but it is also observed for shape-preserving spline. The smoothest impedance plot is given though by fitting the DRT (distribution of relaxation times) model to the measured data (how it is described in [5]):

(12) $$A(t)=a+be^{-{\left(\frac{t}{\tau }\right)}^{\beta }}$$

In this case, three parameters were fitted (b,β,τ),since it was possible to determine offset 𝑎 from the late time data when the relaxation is already over. In general, the more parameters we fit the more precise the result can be. However, computation time increases as well as (in many cases) the sensitivity with respect to noise since the more detailed the fit is the more noise effects we explain.

**Fig. 7:**
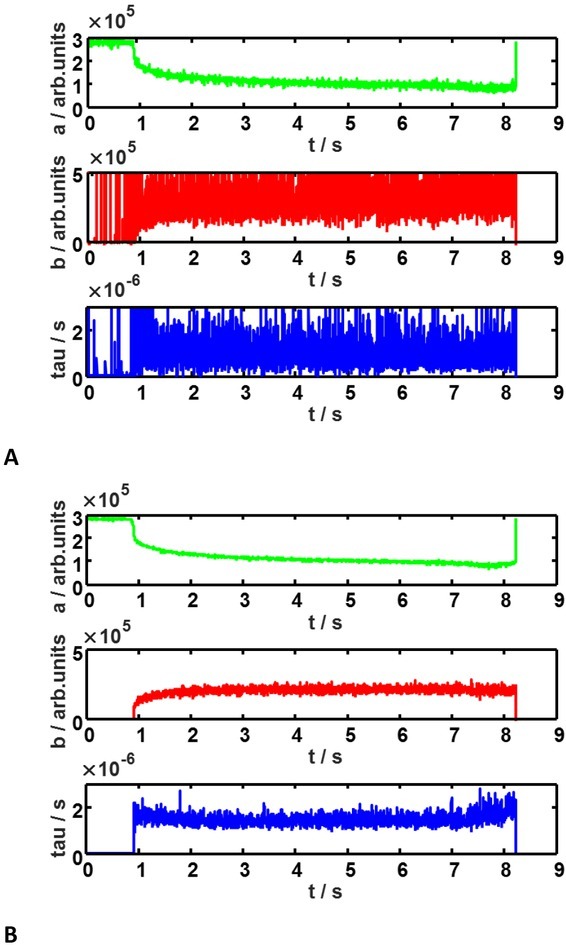
Model parameters after fitting the measured electrical signals for spheroid in the chamber. **A**: simple fit of the distribution of relaxation times model and **B**: the same model fit with proposed improvements.

We can see it in figure 7 how the estimate of the model parameters in two-parameter (b,τ)fit is improved by weighting the objective function according to eq. 11 and the other described steps.

The spheroid appeared about t=0.9 s and it was kept in the chamber for approximately seven seconds for test purposes.

**Fig. 8:**
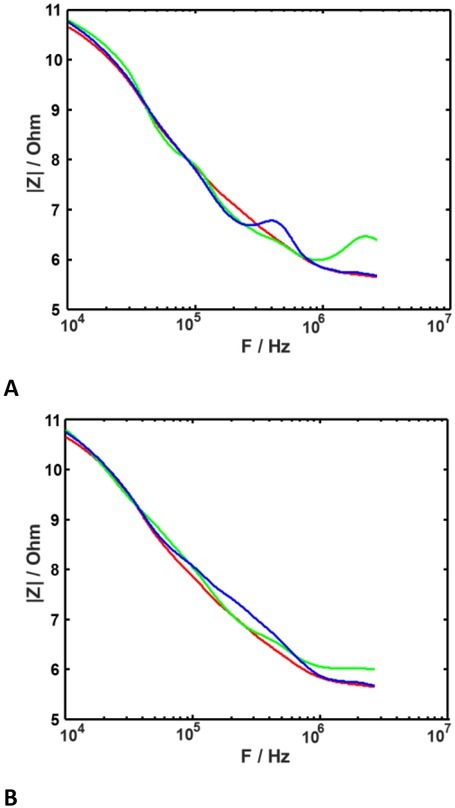
Local signal approximations for spheroid in nozzle. **A**: without correction. **B**: with correction.

Let us now have a look at how the ‘’monotonizing’’ algorithm is working (figure 8). Note, that such a procedure does not modify the measured data in case a priori conditions were not violated due to random perturbations. Such a case corresponds to the red curve in figure 8. The other two curves were modified so that artifact disappeared.

## Conclusion

Previously in [5], a minimalistic impedimetric time-domain measurement system with non-uniform sampling was introduced. In this paper corresponding data processing is discussed. We have proposed and analyzed several methods that help to reconstruct impedance of biological objects from their step response. We show different ways of how it is practically possible to take into account a priori information about the measured signals and how it helps to improve the impedance measurements. The presented algorithms are straightforward and simple enough for realization in microcontrollers.
